# Parafoveal Processing of Orthographic, Phonological, and Semantic Information from Chinese Characters at a Distant Position: A Parafoveal Priming Study

**DOI:** 10.3390/bs15111584

**Published:** 2025-11-19

**Authors:** Xiaoyuan Yuan, Sainan Li, Guoli Yan

**Affiliations:** 1 Faculty of Psychology, Tianjin Normal University, Tianjin 300387, China; yxypsy@stu.tjnu.edu.cn; 2 Key Research Base of Humanities and Social Sciences of the Ministry of Education, Academy of Psychology and Behavior, Tianjin Normal University, Tianjin 300387, China; 3 Tianjin Academy of Educational Sciences, Tianjin 300191, China; snli0206@outlook.com

**Keywords:** parafoveal processing, Chinese, orthography, phonology, semantics

## Abstract

Previous research has shown that the perceptual span in Chinese reading extends three characters to the right of the fixation point. However, little is known regarding the types of preview information that can be extracted from such a distant position; namely, the character at the N + 3 position. Using the parafoveal priming paradigm combined with eye-tracking technology, we manipulated the preview type and preview duration to examine whether Chinese readers could extract orthographic, phonological, and semantic information from the character at the N + 3 position across three experiments. Experiment 1 revealed an orthographic preview cost: orthographically similar previews delayed the target character recognition compared to unrelated previews. Experiment 2 showed no evidence of phonological preview effects. Experiment 3 demonstrated a semantic preview benefit: semantically related previews significantly facilitated the target character recognition relative to unrelated previews. Taken together, these findings indicate that Chinese readers are able to extract orthographic and semantic, but not phonological, information from a distant parafoveal position.

## 1. Introduction

During reading, readers make eye movements to bring new information into the fovea, the region of highest visual acuity, which spans the central 2° of the visual field (Rayner, 1998). Visual acuity declines rapidly with the increase in distance from fixation in the parafoveal region (which spans 2°–5° of the visual field). Despite reduced acuity, readers can still extract information from the parafoveal region, a process known as parafoveal preview (Rayner, 2009).

Previous research on parafoveal preview has primarily focused on two issues: first, the range of useful information readers can extract during a fixation, also referred to as the perceptual span (McConkie & Rayner, 1975), and second, what types of preview information—orthographic, phonological, or semantic—readers can extract from the parafovea (Schotter et al., 2012). Eye-movement studies have shown that the perceptual span, in Chinese—where the character is the basic written unit—extends one character to the left and three characters to the right of fixation (Inhoff & Liu, 1998). Most research on preview types has examined the first or second character to the right of fixation (i.e., characters at the N + 1 or N + 2 position). However, it remains unclear whether readers can also effectively obtain orthographic information, and possibly higher-level phonological or semantic information, from the third character to the right of fixation (i.e., the character at the N + 3 position). The present study was designed to address this issue.

### 1.1. Parafoveal Preview in Alphabetic Writing Systems

In alphabetic writing systems, multiple studies have investigated the extraction of orthographic, phonological, and semantic information from words within the perceptual span (Hermena et al., 2021; Milledge et al., 2022; see also Schotter et al., 2012, for a review).

From the first word to the right of fixation (i.e., word N + 1), studies have shown that readers can extract orthographic information (Johnson et al., 2007; Milledge et al., 2021, 2022; Rayner et al., 1986) as well as phonological information (Ashby et al., 2006; Chace et al., 2005; Miellet & Sparrow, 2004; Milledge et al., 2022). However, there remains considerable debate regarding whether readers can obtain semantic information during reading (Y. Pan et al., 2024).

Rusich et al. (2020) employed a rapid parallel visual presentation (RPVP) paradigm to investigate parafoveal semantic preview in Italian. Two words were presented simultaneously in this paradigm, with one in the foveal region and the other in the parafoveal region. By manipulating the semantic relatedness between the two words, they found that readers were able to extract semantic information from word N + 1. Building on this, Primativo et al. (2022) further demonstrated that this semantic preview effect occurs within a very short time window, with semantic information being activated within 100 ms of parafoveal word presentation. Hohenstein and Kliegl (2014) also reported semantic preview effects in German using a boundary paradigm, showing that readers were able to extract semantic information from word N + 1 by comparing semantically related and unrelated previews. In contrast, Rayner et al. (2014), employing the same paradigm, found no evidence of semantic preview effects in English. This discrepancy could be explained by cross-linguistic differences in orthographic depth. Compared to Italian and German, English has a deeper orthography (Ziegler & Goswami, 2005), which demands greater resources for phonological decoding and consequently leaves fewer resources available for higher-level semantic processing from the upcoming word (Schotter, 2013).

For the second word to the right of fixation (i.e., word N + 2), meta-analytic evidence suggests that readers can extract preview information (Vasilev & Angele, 2017). Some studies have shown that when the word N + 1 is short (e.g., a three-letter word), readers are able to effectively extract preview information from word N + 2 (Kliegl et al., 2007; Radach et al., 2013). In contrast, other studies have reported no preview effects from word N + 2 (Angele et al., 2008; Altarriba et al., 2001; Vasilev & Angele, 2017), indicating that word length is an important factor influencing parafoveal preview. In addition, Cutter et al. (2014) investigated parafoveal processing of word N + 2 using compound words (e.g., teddy bear) as parafoveal stimuli. They found that readers were able to extract information from the second constituent (e.g., bear). This effect is thought to arise because the two constituents of a compound word can be represented as a single unit in the mental lexicon and processed as an integrated whole during sentence reading, thereby allowing readers to obtain a preview benefit from word N + 2.

### 1.2. Parafoveal Preview in Chinese

Findings from alphabetic writing systems cannot be directly generalized to Chinese, given its unique visual and linguistic characteristics. First, Chinese is unspaced and visually dense, with square-shaped characters. Second, as a logographic writing system, Chinese exhibits a relatively high degree of semantic transparency. These characteristics may facilitate the extraction of parafoveal information, particularly higher-level semantic information (Hoosain, 1991). This raises an important question: What types of information can Chinese readers extract from the parafoveal region within their perceptual span?

Previous studies have shown that Chinese readers can extract certain types of information from parafoveal characters within their perceptual span (Schotter et al., 2012; Vasilev & Angele, 2017). Given that the perceptual span in Chinese reading is typically defined at the character level, and each character simultaneously conveys orthographic, phonological, and semantic information, the following section summarizes the types of information that readers can obtain from parafoveal characters within the perceptual span.

Regarding the first character to the right of fixation (i.e., character N + 1), there is substantial evidence that Chinese readers can extract orthographic (Liu et al., 2002; Zhang et al., 2024), phonological (Liu et al., 2002; Pollatsek et al., 2000; J.-L. Tsai et al., 2004), and semantic information (Yan et al., 2009; J. Tsai et al., 2012; Zhang et al., 2024).

Findings regarding the second character to the right of fixation (i.e., character N + 2) are more mixed. Some studies have demonstrated that readers can extract information from this position (Yan et al., 2010; Yang et al., 2012), whereas others suggest that preview effects emerge only when the character N + 1 is of high frequency (Yang et al., 2009; Yang et al., 2012), indicating that parafoveal processing of N + 2 is conditional. Further work has examined the type of information available at this position. For example, Bai et al. (2015) showed that when character N + 2 was orthographically similar rather than dissimilar to character N + 1, fixation durations on character N + 1 were shorter, suggesting that Chinese readers can extract orthographic information from character N + 2.

Most research on the third character to the right of fixation (i.e., character N + 3) has examined parafoveal processing in the context of multi-constituent units such as idioms. Zang (2019) proposed the Multi-Constituent Unit (MCU) Hypothesis, which argues that certain multi-word expressions, such as idioms, may be lexicalized and represented as a single lexical representation in the mental lexicon. For instance, the frequently used Chinese idiom 铁饭碗 (literally “iron rice bowl”, meaning “a secure job”) (Zang et al., 2024a) illustrates this point. Consistent with this account, Zang et al. (2021) used three-character idioms as parafoveal preview stimuli and found that readers could extract information from the third character (e.g., 碗 in 铁饭碗). Zang et al. (2024b) similarly employed four-character idioms (e.g., 风雨同舟, literally “sharing a boat in stormy weather”, meaning “stand together through hardships”) and reported that readers could successfully preview information from the final constituent (e.g., 同舟).

The above findings provide strong evidence that Chinese readers can extract parafoveal information from as far as the third or even fourth character to the right of fixation. However, these preview effects have been observed primarily in idioms or other highly lexicalized multi-character units, suggesting that the benefit may depend heavily on lexicalized structure or familiarity. Moreover, most prior studies have focused simply on whether readers could obtain parafoveal information from distant parafoveal characters, without clarifying the specific types of information involved. Consequently, it remains unclear whether readers can reliably extract orthographic, phonological, and semantic information from Chinese characters at a more distant position (e.g., character at the N + 3 position). Addressing this question is therefore essential for advancing our understanding of the mechanisms underlying parafoveal preview processing.

### 1.3. Parafoveal Priming Study

A variety of experimental paradigms have been developed to investigate parafoveal preview. Some studies have examined preview effects during sentence reading and demonstrated that these effects can be influenced by sentence-specific characteristics. On the one hand, the semantic plausibility of a sentence can affect the extraction of parafoveal information. For example, Schotter and Jia (2016) reported that semantic preview effects only emerged under semantically plausible conditions, raising the question of whether the observed effects reflect genuine semantic pre-processing or merely predictions based on context (Rusich et al., 2020). On the other hand, the processing demands of other words in a sentence can also modulate preview effects. For example, Yang et al. (2009, 2012) reported that preview effects at word N + 2 only occurred when word N + 1 was high-frequency, suggesting that a relatively low processing load is a prerequisite for extracting information from parafoveal words.

Some studies have employed the parafoveal priming paradigm to investigate parafoveal preview (Khelifi et al., 2015; Kwantes & Mewhort, 2002; Pollatsek et al., 2000; Rayner et al., 1978; Veldre et al., 2023). In this paradigm, a preview word is briefly presented in the parafoveal region before the target word appears, and the preview can be orthographically, phonologically, or semantically similar or dissimilar to the target. A related preview influencing processing of the target compared with an unrelated preview, as reflected in reaction times or accuracy, indicates that information from the preview word has been partially pre-processed. 

Snell et al. (2018) further validated the effectiveness of this paradigm in two experiments. In a sentence reading task (Experiment 1), no semantic preview effects were observed. However, in a lexical decision task using the parafoveal priming paradigm (Experiment 2), significant semantic priming effects were found, suggesting that readers can extract parafoveal semantic information when words are presented outside of a sentence context. 

Pollatsek et al. (2000) applied this paradigm to investigate parafoveal processing of Chinese characters. Their results indicated that Chinese readers could extract orthographic and phonological information from parafoveal characters but not semantic information. However, as noted by Pollatsek et al. (2000), the study had two limitations. First, the preview characters were presented at a relatively large eccentricity (4.7° to the right of fixation), which may have attenuated the preview effects due to reduced visual acuity. Second, participants had to deliberately shift their gaze to the parafoveal region, making it difficult to precisely control the preview duration for each character. Given that the preview duration is a critical factor affecting parafoveal processing (Hohenstein et al., 2010; J. Pan et al., 2020), the results of Pollatsek et al. (2000) may have underestimated Chinese readers’ ability to extract information from parafoveal characters.

### 1.4. The Current Study

In summary, research on parafoveal processing of distant Chinese characters and the types of information that readers can extract remains in its early stages. Visual acuity drops off rapidly in the parafoveal region as distance from fixation increases, resulting in substantial reductions in both processing efficiency and word recognition accuracy (Veldre et al., 2023). This raises the question of whether readers’ visual acuity and attentional resources are sufficient to support the extraction of orthographic, phonological, and semantic information from more distal parafoveal positions.

A parafoveal priming paradigm combined with a single-character lexical decision task and eye-tracking was employed in the present study to investigate readers’ parafoveal processing ability independently of sentence context and plausibility. Across three experiments, we examined whether Chinese readers could extract orthographic, phonological, and semantic information from a more distal parafoveal position (i.e., character at the N + 3 position). By presenting stimuli outside of sentence context, the parafoveal priming paradigm provides a relatively “pure” measure of parafoveal processing, laying the groundwork for understanding the mechanisms underlying preview effects in natural reading.

The position of preview characters was carefully controlled in the present study based on the objectives and methodological considerations outlined above. To ensure that parafoveal preview fell within the perceptual span, the preview character was presented at the position corresponding to the third character to the right of the fixation in a sentence; i.e., at the N + 3 position. Specifically, the distance from the center of fixation to the left edge of the preview character was set to span precisely two and a half characters. In eye-tracking studies of Chinese reading, each character typically subtends about 1° of the visual angle (Chang et al., 2024; Zhang et al., 2023). Each preview character was constrained to occupy approximately 1° of the visual angle to ensure precise positioning, resulting in 2° of the visual angle between the left edge of the preview character and the right edge of the target word.

Given that the preview duration is a critical factor influencing parafoveal processing (Hohenstein et al., 2010; J. Pan et al., 2020), three exposure durations for preview characters were employed in the present study. First, since parafoveal information extraction can begin as early as 60 ms after stimulus onset (Rayner et al., 2006), 60 ms was selected as the shortest preview duration. Second, considering that saccade latency is approximately 150 ms (Rayner, 1998), the longest preview duration was set to 145 ms to ensure that previews occurred prior to saccade initiation. An intermediate duration of 100 ms was also included to further examine the time course of preview processing.

## 2. Experiment 1

Experiment 1 aimed to examine the orthographic preview effect for Chinese characters at the N + 3 position. Specifically, three preview conditions were employed: identical, orthographically similar, and unrelated. The experiment addressed two main questions. First, can readers extract orthographic information from parafoveal characters? Given that the activation of orthographic representations serves as a prerequisite for the activation of other types of representations, such as phonology or semantics (Perfetti & Tan, 1998; Reichle & Yu, 2018), we hypothesized that readers would be able to extract orthographic information from preview characters even at a distant parafoveal position, as reflected by a significant difference between the orthographically similar and unrelated conditions. Second, what is the time course of orthographic preview—that is the temporal window following stimulus onset during which the preview effect emerges, providing insight into the timing of orthographic information extraction?

### 2.1. Method

#### 2.1.1. Participants

We determined the sample size for this study through an a priori power analysis. Eighteen participants would be required to detect a medium effect size (*f* = 0.25) with 90% statistical power at *α* = 0.05 (G*Power 3.1) (Faul et al., 2009). To ensure robust findings, a total of 36 undergraduate and graduate students participated in Experiment 1, including 18 females and 18 males. The mean age of all the participants was 19.92 years (*SD* = 1.89). All participants were native Chinese speakers with normal or corrected-to-normal vision. Informed consent was obtained from all participants.

#### 2.1.2. Apparatus

Eye movements were recorded using an SR Research Eyelink 1000 eye-tracker with a sampling rate of 1000 Hz. Stimuli were presented in black on a white background on a monitor with a refresh rate of 120 Hz and a resolution of 1024 × 768 pixels. Participants viewed the screen from a distance of 65 cm. Characters were presented in 32-pixel Song font, with each character subtended approximately 1° of the visual angle.

#### 2.1.3. Materials

A total of 180 target characters were selected from the *Dictionary of Orthographically Similar Chinese Characters* (Ran, 2010). Each target character was paired with three types of preview characters—identical, orthographically similar, and unrelated—which were presented to the right of fixation. The character frequency and number of strokes were matched across the three preview conditions using the Subtlex-CH corpus (Cai & Brysbaert, 2010) (see Table 1). No significant differences were found among the three conditions in terms of the character frequency (*F* (2, 178) = 0.08, *p* = 0.92) or number of strokes (*F* (2, 178) = 1.26, *p* = 0.28). Ninety character pairs were included as fillers in which the preview character appeared to the right of fixation, and the target characters were pseudocharacters. To prevent participants from allocating attention exclusively to the right visual field, an additional 136 pairs were used as fillers, with the preview character presented to the left of fixation. Of these, 68 pairs had real characters as targets, and 68 pairs had pseudocharacters as targets. All filler items were excluded from data analysis.

Fifteen undergraduate students who did not take part in the main experiment rated the orthographic similarity between each target character and its orthographically similar or unrelated preview on a five-point Likert scale (1 = very dissimilar, 5 = very similar). The target characters were rated as highly similar to their orthographically similar previews (*M* = 3.90, *SD* = 0.46), but much less similar to the unrelated previews (*M* = 1.36, *SD* = 0.45).

#### 2.1.4. Procedure and Task

At the beginning of each trial, a fixation cross (“+”) first appeared at the center of the screen, and the participants were instructed to maintain their gaze on this position. Once fixation stability was confirmed using the eye tracker, a preview character was presented either to the left or right of fixation in random order for 60, 100, or 145 ms. During this preview period, the participants were required to continue fixating on the central cross. A target character appeared at the center of the screen immediately afterward, and the participants were asked to recognize as quickly as possible whether the character was a real Chinese character by pressing a key.

**Figure 1:**
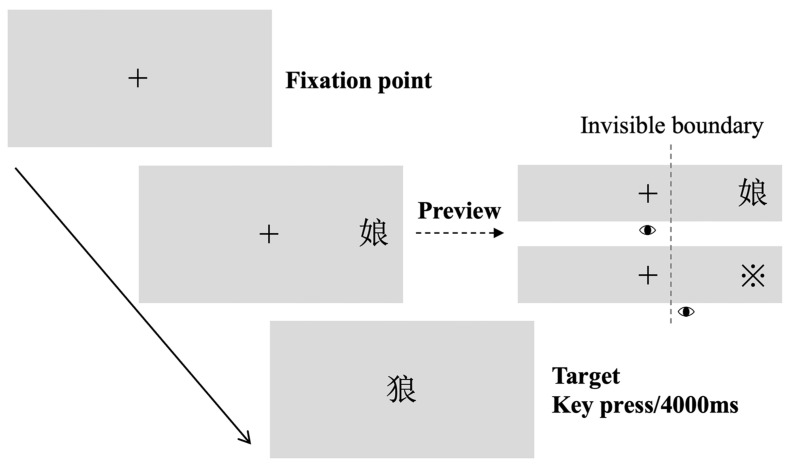
Schematic diagram of the experimental procedure. In this example, the preview was presented in the right parafoveal field.

An invisible boundary was set between the fixation point and the preview character to ensure that the preview character remained at the intended parafoveal position. If the participant’s gaze crossed this boundary during the preview period, the preview character was replaced with the masking symbol (“※”). The experimental procedure is illustrated in Figure 1.

### 2.2. Results

Trials were removed for the following reasons: (a) insufficient preview duration because participants’ eyes deviated from the fixation point (13.1%), (b) reaction times exceeding three standard deviations from the mean (2.5%), and (c) incorrect responses, which were excluded from the reaction time analyses (2.1%).

Linear mixed models (LMMs) were applied using the lme4 package in R (version 4.3.2, R Core Team, 2023) to analyze the data. The preview type and preview duration were treated as fixed effects. Participants and items were included as random effects. Linear mixed-effects models were used to analyze reaction time data, and generalized linear mixed-effects models were used to analyze error rate data. After exclusions, a total of 5394 trials were included in the reaction time analyses, and 5589 trials were included in the accuracy analyses.

#### 2.2.1. Reaction Time (RT)

The main effect of the preview type was significant. RTs were significantly shorter in the identical condition than those in the orthographically similar and unrelated conditions (|*t*|s *>* 7.48, *p*s < 0.01). Moreover, RTs in the orthographically similar condition were significantly longer than in the unrelated condition (*b* = −0.02, *SE* = 0.01, *t* = −2.69, *p* < 0.001). The main effect of the preview duration was also significant, with RTs in the 60 ms preview condition significantly longer than in the 100 and 145 ms preview conditions (|*t*|s *>* 2.87, *p*s < 0.001). No significant difference was found between the 100 and 145 ms preview conditions (*b* = −0.01, *SE* = 0.01, *t* = −1.18, *p* = 0.24). 

The difference in RTs between the orthographically similar and unrelated conditions was taken as the measure of the orthographic preview effect. A significant interaction between the preview type and preview duration was observed, indicating differences in the size or direction of the orthographic preview effect across temporal stages. Simple effects analyses revealed no orthographic preview effect in the 60 ms preview condition as reaction times did not differ between the orthographically similar and unrelated conditions (*b* = 0.004, *SE* = 0.01, *t* = 0.36, *p* = 0.72). In contrast, significant orthographic preview effects emerged in both the 100 and 145 ms preview conditions, with RTs in the orthographically similar condition being significantly longer than in the unrelated condition (|*t*|s > 3.86, *p*s < 0.01). The effect sizes were −19 ms in the 100 ms preview condition and −21 ms in the 145 ms preview condition, both significantly larger than that of the 60 ms condition (4 ms; |*t*|s > 1.85, *p*s < 0.06). No significant difference in effect size was observed between the 100 and 145 ms conditions (*b* = −0.01, *SE* = 0.02, *t* = − 0.54, *p* = 0.59). See Table 2 for means and standard deviations of RTs.

#### 2.2.2. Error Rates

The main effect of the preview type was significant, with higher error rates in the orthographically similar condition than in the identical and unrelated conditions (|*z*|s > 2.54, *p*s < 0.01). Neither the main effect of preview duration (|*z*|s < 1.12, *p*s > 0.26) nor the interaction between the preview type and preview duration was significant (|*z*|s < 0.86, *p*s > 0.39). See Table 3 for means and standard deviations of error rates.

### 2.3. Discussion

The findings of Experiment 1 can be summarized in two main points. First, the results demonstrated that Chinese readers could extract orthographic information from the character at the N + 3 position. Second, the time course of preview processing was further examined by analyzing effects across different preview durations. Stable orthographic preview effects were observed in the 100 ms and 145 ms preview conditions, with effect sizes of −19 ms and −21 ms, respectively, increasing with the preview duration. In contrast, no significant orthographic preview effect was found in the 60 ms preview condition. RTs decreased with longer preview durations, indicating that extended previews facilitated the recognition of the target character.

In addition, the results revealed an orthographic preview cost: orthographically similar previews delayed the target character recognition compared with unrelated previews. This finding is consistent with Pollatsek et al. (2000), who also observed orthographic preview cost using a parafoveal-on-foveal priming paradigm. J. Pan et al. (2020) examined the semantic preview effect in Chinese reading and found that short preview durations (80 ms) facilitated the target character processing for semantically related previews, reflecting a semantic preview benefit. In contrast, longer preview durations (150 ms) or full preview conditions (no time constraint) produced semantic preview costs as semantically related previews interfered with the target character recognition. This effect arises because longer preview durations allow for more in-depth processing of the previewed character, activating more semantics that are inconsistent with the target, thereby inducing competition and reducing processing efficiency (J. Pan et al., 2020). In the present study, the observed orthographic preview cost may similarly have resulted from the longer preview durations (100 ms and 145 ms), which allowed the features of orthographically similar previews to become more strongly activated. These subtle differences from the target character then competed with the target’s orthographic representation, thereby interfering with its recognition.

## 3. Experiment 2

The aim of Experiment 2 was to examine the phonological preview effect for Chinese characters at the N + 3 position. Specifically, three preview conditions were employed: identical, homophonic, and unrelated preview. The experiment addressed two main questions. First, can readers extract phonological information from the parafoveal characters? Evidence for such access would be demonstrated by significant differences between the homophonic and unrelated preview conditions. Second, what is the time course of phonological preview; that is, what is the temporal window following stimulus onset during which the preview effect emerges? We explored this question with the aim of providing insight into the timing of phonological information extraction.

### 3.1. Method

#### 3.1.1. Participants

A total of 36 undergraduate and graduate students participated in Experiment 2, including 19 females and 17 males. The mean age of all the participants was 20.75 years (*SD* = 1.85). All participants were native Chinese speakers with normal or corrected-to-normal vision. Informed consent was obtained from all participants.

#### 3.1.2. Materials

A total of 180 target characters were selected from the *Dictionary of Homophones* (Sun, 2014). Each target character was paired with three types of preview characters—identical, homophonic, and unrelated—which were presented to the right of fixation. The character frequency and number of strokes were matched across the three preview conditions using the Subtlex-CH corpus (Cai & Brysbaert, 2010) (see Table 4)**.** No significant differences were found among the three conditions in terms of the character frequency (*F* (2, 178) = 0.46, *p* = 0.63) or number of strokes (*F* (2, 178) = 0.62, *p* = 0.54). Ninety character pairs were included as fillers, in which the preview character appeared to the right of fixation. In Chinese, the syllable *yi* is among the most homophone-rich phonetic syllables, offering abundant substitution options; therefore, characters pronounced “yi” were selected as target characters in this study. An additional 136 pairs were used as fillers, with the preview character presented to the left of fixation to prevent participants from allocating attention exclusively to the right visual field. Of these, 68 contained target characters pronounced as “yi”. All filler items were excluded from data analysis.

Fifteen undergraduate students who did not take part in the main experiment rated the orthographic similarity between each target character and its homophonic preview on a five-point Likert scale (1 = very dissimilar, 5 = very similar). The target characters were rated to be highly dissimilar to their homophonic previews, *M* = 1.36, *SD* = 0.45.

#### 3.1.3. Procedure and Task

The participants were asked to recognize as quickly as possible whether the target character was pronounced as “yi” (regardless of tone) and respond by pressing a key. The overall procedure of Experiment 2 was similar to that of Experiment 1.

### 3.2. Results

Trials were removed for the following reasons: (a) preview duration was not sufficient because participants’ eyes deviated from the fixation point (8.6%), (b) reaction times exceeding three standard deviations from the mean (1.8%), and (c) incorrect responses, which were excluded from the reaction time analyses (2.0%). After exclusions, a total of 5701 trials were included in the reaction time analyses, and 5922 trials were included in the accuracy analyses.

#### 3.2.1. Reaction Time (RT)

The main effect of the preview type was significant. RTs were significantly shorter in the identical condition than those in the homophonic and unrelated conditions (|*t*|s *>* 7.74, *p*s < 0.01). No significant difference was found between the homophonic and unrelated conditions (*b* = 0.01, *SE* = 0.07, *t* = 1.81, *p* = 0.07). The main effect of the preview duration was also significant, with RTs in the 145 ms preview condition significantly shorter than in the 60 and 100 ms preview conditions (|*t*|s *>* 3.44, *p*s < 0.01), and RTs in the 100 ms preview condition were significantly shorter than in the 60 ms preview condition. The interaction effect was not significant (|*t*|s < 0.46, *p*s > 0.64), indicating no phonological preview effect across all three preview durations. See Table 5 for means and standard deviations of RTs.

#### 3.2.2. Error Rate

The main effects of the preview type and preview duration, as well as their interaction, were all non-significant (*|z|*s < 1.05, *p*s > 0.29). See Table 6 for means and standard deviations of error rates.

### 3.3. Discussion

The results of Experiment 2 showed that, across all three preview durations, RTs and error rates did not significantly differ between the homophonic and unrelated preview conditions. This indicates that Chinese readers were unable to obtain phonological preview information for the character at the N + 3 position. The effect sizes for the three preview durations were 0 ms, 10 ms, and 16 ms, showing a slight trend toward increased facilitation with longer preview durations.

Previous studies have found that the average fixation duration during natural reading is approximately 200 ms (Schotter et al., 2012), which is close to the longest preview duration used in this experiment. Furthermore, during natural reading, readers must simultaneously process other words in the sentence. This limits available processing resources and may constrain the extraction of parafoveal information from more distant locations. Taken together, these findings imply that, even under natural reading conditions, substantial phonological preview from the N + 3 character is unlikely.

## 4. Experiment 3

The aim of Experiment 3 was to examine the semantic preview effect for Chinese characters at the N + 3 position. Specifically, three preview conditions were employed: identical, semantically related, and unrelated preview. The experiment addressed two main questions. First, can readers extract semantic information from parafoveal characters? Evidence for such access would be demonstrated by significant differences between the semantically related and unrelated preview conditions. Second, what is the time course of semantic preview; that is, what is the temporal window following stimulus onset during which the preview effect emerges? We explored this question with the aim of providing insight into the timing of semantic information extraction.

### 4.1. Method

#### 4.1.1. Participants

A total of 36 undergraduate and graduate students participated in Experiment 3, including 19 females and 17 males. The mean age of all the participants was 20.08 years (*SD* = 1.47). All participants were native Chinese speakers with normal or corrected-to-normal vision. Informed consent was obtained from all participants.

#### 4.1.2. Materials

A total of 144 target characters were selected from the *Dictionary of Synonyms, Antonyms, with Example Sentences* (Mei, 2017). Each target character was paired with three types of preview characters—identical, semantically related, and unrelated—which were presented to the right of fixation. The character frequency and number of strokes were matched across the three preview conditions using the Subtlex-CH corpus (Cai & Brysbaert, 2010) (see Table 7). No significant differences were found among the three conditions in terms of the character frequency (*F* (2, 142) = 0.11, *p* = 0.89) or number of strokes (*F* (2, 142) = 0.10, *p* = 0.91). Seventy-two character pairs were included as fillers in which the preview character appeared to the right of fixation, and the target characters were represented as animals (e.g., dog). To prevent participants from allocating attention exclusively to the right visual field, an additional 108 pairs were used as fillers, with the preview character presented to the left of fixation. Of these, 54 contained target characters represented as animals. All filler items were excluded from data analysis.

Fifteen undergraduate students who did not take part in the main experiment rated the semantic similarity between each target character and its semantically related preview on a five-point Likert scale (1 = very dissimilar, 5 = very similar). The target characters were rated to be highly similar to their semantically related previews, *M* = 4.28, *SD* = 0.34.

#### 4.1.3. Procedure and Task

The participants were asked to recognize as quickly as possible whether the target character represented an animal and respond by pressing a key. The overall procedure of Experiment 3 was similar to that of Experiment 1.

### 4.2. Results

Trials were removed for the following reasons: (a) preview duration was not sufficient because participants’ eyes deviated from the fixation point (12.2%), (b) reaction times exceeding three standard deviations from the mean (1.3%), and (c) incorrect responses, which were excluded from the reaction time analyses (0.9%). After exclusions, a total of 4393 trials were included in the reaction time analyses, and 4437 trials were included in the accuracy analyses.

#### 4.2.1. Reaction Time (RT)

The main effect of the preview type was significant. RTs were significantly shorter in the identical condition than in the semantically related and unrelated conditions (|*t*|s *>* 3.12, *p*s < 0.01), and RTs in the semantically related condition were significantly shorter than those in the unrelated condition (*b* = 0.04, *SE* = 0.01, *t* = 4.32, *p* < 0.01). Neither the main effect of the preview duration (|*t*|s < 1.50, *p*s > 0.13) nor the interaction between the preview type and preview duration (|*t*|s < 0.56, *p*s > 0.58) was significant, indicating that a robust semantic preview benefit was observed across all three preview durations. See Table 8 for means and standard deviations of RTs.

#### 4.2.2. Error Rate

The main effects of the preview type (*|z|*s < 1.10, *p*s > 0.27) and preview duration (*|z|*s < 0.54, *p*s > 0.68), as well as their interaction (*|z|*s < 0.28, *p*s > 0.78), were all non-significant. See Table 9 for means and standard deviations of error rates.

### 4.3. Discussion

Experiment 3 showed that Chinese readers can extract semantic information from the character at the N + 3 position across all preview durations. The semantic preview effect measured 22 ms, 28 ms, and 28 ms across three preview durations, indicating an initial increase that quickly reached a plateau. In addition, RTs decreased as the preview duration increased, suggesting that longer previews facilitated the target character recognition.

These findings suggest that readers can engage in higher-level semantic processing of distant parafoveal characters, even without supportive sentence context. This contrasts with the more limited semantic preview effects often reported in alphabetic languages (Altarriba et al., 2001; Rayner et al., 2014), suggesting a potential advantage of the Chinese writing system for extracting semantic information.

## 5. Discussion

Eye-tracking with a parafoveal preview paradigm and a single-character decision task were employed in this study to investigate orthographic, phonological, and semantic information for characters at a more distant parafoveal position (i.e., the character at the N + 3 position). By varying the preview type and duration, we measured reaction times and error rates in the target character recognition. The findings show that readers were able to extract orthographic and semantic information from the character at the N + 3 position, but not phonological information.

### 5.1. Orthographic, Phonological, and Semantic Preview Effects

Across the three experiments, identical previews consistently yielded the shortest response times and lowest error rates, indicating that participants can extract useful information from characters at the N + 3 position. Experiment 1 revealed an orthographic preview cost as orthographically similar previews interfered with target recognition compared to unrelated previews, demonstrating that readers can extract orthographic information from the N + 3 position. In contrast, Experiment 2 showed no difference between homophonic and unrelated previews, suggesting that phonological information is not extracted from this more distant position. Finally, Experiment 3 demonstrated a semantic preview benefit, with semantically related previews facilitating target recognition relative to unrelated previews, indicating that readers can extract semantic information from the N + 3 position.

The extraction of orthographic information reflects a key component of the cognitive processes involved in Chinese character recognition. Activation of orthographic representations serves as a prerequisite for subsequent activation of phonological and semantic representations (Perfetti & Tan, 1998; Reichle & Yu, 2018). Because orthographic information is encoded directly through visual input, it can be accessed more readily than phonological or semantic information, even from distal parafoveal locations where visual acuity is substantially reduced.

It was found in this study that readers could extract semantic information but not phonological information from the N + 3 position, a pattern consistent with previous findings. Prior work has also shown that, in Chinese reading, semantic information can often be accessed directly without reliance on phonology (e.g., Chen & Shu, 2001; Yan et al., 2009; X. Zhou & Marslen-Wilson, 1999). For example, Chen and Shu (2001) examined orthographic, phonological, and semantic processing of foveal characters and reported robust semantic preview effects across three preview durations (43 ms, 57 ms, and 85 ms), whereas phonological preview effects were weak and only emerged at 57 ms.

The tendency of Chinese readers to extract semantic rather than phonological information from parafoveal characters arises from the unique features of the Chinese writing system. As a logographic system, Chinese characters exhibit a strong link between orthography and semantics, facilitating direct access to semantics (Yan et al., 2009; J. Tsai et al., 2012). Moreover, Chinese has a relatively deep orthography, with low consistency between orthography and phonology. Many orthographic similar characters have different phonemes, making rapid phonological activation from visual forms difficult (Hoosain, 1991). 

Alphabetic writing systems have shallower orthographic depth than Chinese and provide a stronger link between orthography and phonology, allowing phonology to be more easily accessed than semantics (Verhoeven & Perfetti, 2022). This orthographic transparency may explain why parafoveal preview studies in alphabetic scripts have consistently reported robust phonological preview effects (Ashby et al., 2006; Chace et al., 2005), whereas semantic preview effects tend to vary across languages. For instance, Italian exhibits highly consistent grapheme–phoneme correspondences, enabling readers to rapidly extract phonological information while retaining sufficient cognitive resources to activate semantic representations, resulting in semantic preview effects (Primativo et al., 2022; Rusich et al., 2020). By contrast, the less consistent grapheme–phoneme mappings in English require readers to allocate more cognitive resources to phonological processing, making parafoveal semantic processing more demanding and leading to weaker or more variable semantic preview effects (Altarriba et al., 2001; Rayner et al., 2014).

### 5.2. The Time Course of Orthographic, Phonological, and Semantic Information Processing

Across the three experiments, orthographic preview costs emerged at the 100 ms preview duration, whereas semantic preview benefits were observed as early as 60 ms. No phonological preview effects were detected at any of the three preview durations. These results suggest that semantic information may be activated earlier than orthographic information, a pattern that contrasts with previous findings in Chinese character recognition. Previous studies have generally indicated that orthographic information, as a lower-level component in Chinese character processing, is typically activated before phonological and semantic information (Perfetti & Tan, 1998; Reichle & Yu, 2018). This raises the question of whether semantic activation could precede orthographic activation.

The seemingly contradictory findings can be explained by the temporal dynamics of orthographic preview. The results from Experiment 1 showed that at a 60 ms preview duration, the orthographically similar condition did not differ significantly from the unrelated condition, but a slight facilitation trend was already observable (effect size = 4 ms). As the preview duration increased, the orthographic preview effect shifted from facilitation to a cost, becoming a clear orthographic preview cost at 100 ms. This dynamic pattern aligns with the findings of J. Pan et al. (2020), who investigated the semantic preview effect in Chinese with preview durations of 80 ms, 100 ms, and 150 ms and a full preview condition. They found that semantically related previews facilitated target word processing relative to unrelated previews at 80 ms, but this benefit disappeared at 100 ms. At 150 ms and in the full preview condition, a semantic preview cost was observed.

This shift from facilitation at short preview durations to cost at longer durations can be explained by the Chinese Reading Model (CRM) (Li & Pollatsek, 2020), which integrates word processing and eye-movement control in Chinese reading. The model comprises two modules: a word processing module and an eye-movement control module. The word processing module, based on the Interactive Activation Model (IAM) (McClelland & Rumelhart, 1981), includes three levels: visual, character, and word. Activation and inhibitory connections exist both within and between these levels. At the character level, the activation of orthographic information simultaneously activates multiple orthographically similar candidates, which compete and inhibit one another. For example, at short preview durations, the activation of an orthographically similar preview (e.g., 叫, meaning call) is still in its initial stage: readers recognize only the radical “口” and the approximate structure of the right-side component. Such partial activation can facilitate recognition of the target character (e.g., 听, meaning listen). As the preview duration increases, the right-side component of the similar character becomes more fully processed, leading to stronger competition with the target character and interfering with its recognition, thereby producing a preview cost.

We propose that readers begin decoding the orthographic information of parafoveal characters even before 100 ms, and potentially as early as 60 ms. The orthographic preview effect initially manifests as a facilitation, which gradually diminishes with the increase in the preview duration and ultimately converts into a preview cost at 100 ms. Thus, although our results showed that semantic information produces a benefit at 100 ms, the overall processing sequence in Chinese character recognition still prioritizes orthographic information.

### 5.3. Theoretical Implications for Chinese Character Recognition

Two main theoretical perspectives have been proposed regarding the time course of orthographic, phonological, and semantic activation in alphabetic writing systems. The Phonological Mediation Model posits that orthographic information activates the corresponding phonological information first, which in turn facilitates access to semantic information. Phonology thus serves as a critical bridge between orthographic and semantic processing (Rubenstein et al., 1971). In contrast, the Direct Access Model argues that orthography can directly activate semantics, and phonology is not necessary for accessing semantics (Taft & van Graan, 1998). X. L. Zhou (1997) proposed a model of character representation and processing for Chinese in which orthography forms the foundation of character recognition. Once orthography is activated, semantics and phonology can be accessed in parallel, resulting in a time course of activation that differs from alphabetic scripts.

The results of the present study indicate that Chinese readers exhibited semantic preview benefits but no phonological preview effects. This pattern suggests that semantic information can be accessed directly, without mediation by phonological activation, providing support for the Direct Access Model and offering empirical validation for X. L. Zhou’s (1997) framework of Chinese character processing.

However, another possible explanation should also be considered. Perfetti et al. (1992) proposed the universal phonological principle, suggesting that phonological processing is involved in reading across all writing systems, although the form and timing of this processing may vary depending on the orthography. Similarly, in his blueprint for the reader, Perfetti (1999) argued that phonological information is activated even in logographic systems such as Chinese. Therefore, it is possible that in the present study, phonological activation was still accumulating and did not emerge within the temporal window captured by the current parafoveal preview paradigm. Future studies could test this hypothesis by extending the preview duration of parafoveal stimuli or employing techniques with higher temporal resolution, such as combined eye-tracking and EEG recordings.

### 5.4. Practical Implications for Chinese Reading Instruction

The present findings suggest that Chinese reading instruction should not only emphasize the sequential mappings from orthography to phonology to semantics but also give particular attention to the direct orthography–semantics pathway.

In particular, instructional approaches that emphasize the visual forms of characters to support semantic access may enable children with reading difficulties arising from phonological processing deficits to recognize words without relying on phonological decoding, thereby enhancing overall reading comprehension and reading efficiency.

Nevertheless, as the participants in the present study were adult readers, these pedagogical implications remain tentative and should be further validated in studies involving child populations.

### 5.5. Limitations

During natural sentence reading, word processing times can approach zero, especially for the words that are highly predictable from the sentence context and are short or easy to process (Balota et al., 1985). In the present study, the parafoveal preview paradigm controlled for the absence of Chinese characters at the foveal N position as well as the parafoveal N + 1 and N + 2 positions, effectively simulating an extremely low processing load at these positions. This design allowed for an ideal examination of whether Chinese readers can extract preview information from a distant parafoveal position. While this paradigm offers a clear advantage in parafoveal processing, it also raises concerns regarding external validity.

Future research could extend these findings by investigating parafoveal preview processing at more distal locations (e.g., the N + 3 position) under natural sentence reading conditions. Sentence reading tasks that manipulate word frequency or predictability from N to N + 2 could help to clarify how these factors influence orthographic, phonological, and semantic preview processing at more distal parafoveal positions.

## 6. Conclusions

In conclusion, orthographic, phonological, and semantic preview processing for characters at a more distal parafoveal position (i.e., the character at the N + 3 position) were investigated in this study. Our results demonstrated that readers were able to extract orthographic and semantic information, but not phonological information, from characters at the N + 3 position. These findings enrich our understanding of parafoveal processing within the Chinese perceptual span and provide new insights into the time course of parafoveal character recognition, highlighting the differential accessibility of orthographic, phonological, and semantic information in Chinese reading.

## Figures and Tables

**Table 1:** The frequency and stroke number of the three preview characters in Experiment 1 *M* (*SD*).

	Identical	Orthographically Similar	Unrelated
Character	狼	娘	针
Meaning	wolf	mother	needle
Pronunciation	lang2	niang2	zhen1
Frequency	110.40 (593.64)	86.98 (419.10)	98.79 (603.43)
Stroke number	9.32 (2.65)	9.28 (2.58)	9.91 (6.27)

**Table 2:** Means and standard deviations of reaction time (*ms*) in Experiment 1 *M* (*SD*).

Preview Type	Preview Duration		
	60	100	145
Identical	609 (157)	583 (167)	563 (163)
Orthographically similar	618 (154)	632 (168)	628 (176)
Unrelated	622 (165)	613 (161)	607 (166)

**Table 3:** Means and standard deviations of error rate in Experiment 1 *M* (*SD*).

Preview Type	Preview Duration		
	60	100	145
Identical	1.52 (0.12)	0.62 (0.08)	1.86 (0.14)
Orthographically similar	2.42 (0.15)	2.87 (0.17)	2.92 (0.17)
Unrelated	1.20 (0.11)	2.17 (0.15)	1.60 (0.13)

**Table 4:** The frequency and stroke number of the three preview characters in Experiment 2 *M* (*SD*).

	Identical	Homophonic	Unrelated
Character	冬	东	胃
Meaning	winter	east	stomach
Pronunciation	dong1	dong1	wei4
Frequency	88.40 (644.91)	151.95 (787.17)	105.39 (485.47)
Stroke number	9.51 (2.65)	9.21 (3.70)	9.39 (2.36)

**Table 5:** Means and standard deviations of reaction time (*ms*) in Experiment 2 *M* (*SD*).

Preview Type	Preview Duration		
	60	100	145
Identical	658 (214)	633 (238)	591 (202)
Homophonic	671 (214)	659 (223)	641 (213)
Unrelated	671 (204)	669 (241)	657 (213)

**Table 6:** Means and standard deviations of error rate in Experiment 2 *M* (*SD*).

Preview Type	Preview Duration		
	60	100	145
Identical	2.45 (0.15)	1.76 (0.13)	1.76 (0.13)
Homophonic	1.91 (0.14)	2.05 (0.14)	2.46 (0.16)
Unrelated	1.85 (0.13)	1.91 (0.14)	2.28 (0.15)

**Table 7:** The frequency and stroke number of the three preview characters in Experiment 3 *M* (*SD*).

	Identical	Semantically Related	Unrelated
Character	晚	迟	短
Meaning	late	late	short
Pronunciation	wan3	chi2	duan3
Frequency	253.91 (709.68)	309.38 (1190.74)	277.38 (971.43)
Stroke number	8.96 (2.85)	8.90 (3.00)	9.03 (2.64)

**Table 8:** Means and standard deviations of reaction time (*ms*) in Experiment 3 *M* (*SD*).

Preview Type	Preview Duration		
	60	100	145
Identical	646 (205)	625 (179)	613 (197)
Semantically related	647 (188)	643 (205)	639 (209)
Unrelated	669 (206)	671 (212)	667 (229)

**Table 9:** Means and standard deviations of error rate in Experiment 3 *M* (*SD*).

Preview Type	Preview Duration		
	60	100	145
Identical	1.19 (0.11)	1.11 (0.10)	1.67 (0.12)
Semantically related	0.33 (0.10)	0.51 (0.10)	0.24 (0.10)
Unrelated	0.49 (0.09)	0.52 (0.10)	0.46 (0.09)

## Data Availability

Data and Study materials can be found on the OSF at the following link: https://osf.io/e9qjt/ (accessed on 23 August 2025).
